# Potential diagnostic and prognostic marker dimethylglycine dehydrogenase (DMGDH) suppresses hepatocellular carcinoma metastasis *in vitro* and *in vivo*

**DOI:** 10.18632/oncotarget.8927

**Published:** 2016-04-22

**Authors:** Gang Liu, Guojun Hou, Liang Li, Yixue Li, Weiping Zhou, Lei Liu

**Affiliations:** ^1^ Key Laboratory of Systems Biology, Institute of Biochemistry and Cell Biology, Shanghai Institutes for Biological Sciences, Chinese Academy of Sciences, Shanghai, China; ^2^ The Third Department of Hepatic Surgery, Eastern Hepatobiliary Surgery Hospital, Second Military Medical University, Shanghai, China; ^3^ International Co-operation Laboratory on Signal Transduction, Eastern Hepatobiliary Surgery Institute, Second Military Medical University, National Center for Liver Cancer, Shanghai, China; ^4^ Institute of Biomedical Sciences, Fudan University, Shanghai, China

**Keywords:** hepatocellular carcinoma, diagnosis, prognosis, metastasis, DMGDH

## Abstract

Key metabolic enzymes regulatethe fluxes of small compounds to provide the basal substrates for cellular architecture and energy. Some of them are reported to be important carcinogenesis- and metastasis-related genes. In our work, we performed RNA-seq for50 pairs of normal-tumor of hepatocellular carcinoma (HCC) samples and found that the expression of dimethylglycine dehydrogenase (DMGDH) is decreased in HCC. The analysis of protein levels with Western blotting and immunohistochemistry also conformed our findings. It is proven to be a valuable biomarker for both diagnosis and prognosis in three independent datasets. Furthermore, we revealed that DMGDH suppresses migration, invasion and metastasis both *in vitro* and *in vivo*. By utilizing gene expression microarray for DMGDH, we identified several possible pathways altered in a DMGDH over-expressing cell line. Among these pathways, we noted that the phosphorylation of Akt-308/473 was significantly suppressed when DMGDH was over-expressed. In summary, our work reveals that DMGDH is a potential valuable biomarker for both diagnosis and prognosisfor HCC, and DMGDH gene expression suppresses metastasis through the Akt signaling pathway.

## INTRODUCTION

Hepatocellular carcinoma (HCC) is the fifth most common cancer and is the third leading cause of death worldwide [1], with 500,000 peopleaffected every year [2]. Furthermore, HCC is a relatively aggressive cancer type, which is characterized by rapid metastasis and development. The high metastasis leads to less than 10% five year survival rate [3], makes it difficult for therapy. Thus, revealing the mechanisms of HCC pathogenesis is necessary for therapy.

Recently, metabolic alterations are reported [4, 5]. By metabolic study, step-limiting enzymes, which are key regulators of metabolic fluxes, have also been implicated. Recently, alteration of important enzymes involved in glucose metabolism including somatic mutation of IDH1/2 in have been reported in HCC patients [6]. In breast cancer, over-expression of PKM2 leads to glucose fluxes into the pentose phosphate pathway (PPP) instead of glycolysis [7], and produce more ribose, which is one of the most important architectural components of DNA and RNA. In HCC, glutaminase 2 was reported to regulate PI3K/AKT signaling pathway and suppress tumor activity [8]. Defects of key enzymes involved in lipid metabolism, including SCD-CoA [9], and enzymes in choline activation werealso reported [10], indicating that metabolic dysfunction contributes to hepatocellular carcinogenesis and development. However, current metabolic biomarker is still insufficient for diagnosis and prognosis in HCC.

In this vein, we performed RNA sequencing on HCC patients, and detected that dimethylglycine dehydrogenase (DMGDH) decreased in HCC tissue compared with the corresponding normal tissue. In addition, the proportion of somatic mutation and/or loss of heterogeneity of this gene reached 36% in our dataset. The overall survival rate of patients with higher DMGDH expression was significantly higher than those with lower levels. We then validated these findings with public data from TCGA dataset, another dataset, and immunohistochemical analysis. We knocked down and over-expressed DMGDH in twodifferent HCC cell lines. Although the proliferation rate was relatively stable in both cell lines before/after knock-down/over-expression, cells with lower DMGDH expression exhibited higher motility. We then constructed a mouse lung metastasis model, and found that metastasis withinthe DMGDH over-expression group was significantly slower than the normal group. We also evaluated gene expression levels of both the DMGDH and control groups by microarray. Ingenuity Pathway Analysis (IPA) of the differentially expressed genes between the two groups revealed that several pathways were altered, including the PI3K/Akt signaling pathway. Western blot results demonstrated that over expression of DMGDH causes a decreased phosphorylation of Akt-308/473, which in turn suppresses metastasis.

## RESULTS

### DMGDH is significantly altered in tumors and is a potential biomarker for HCC diagnosis

**Figure 1:**
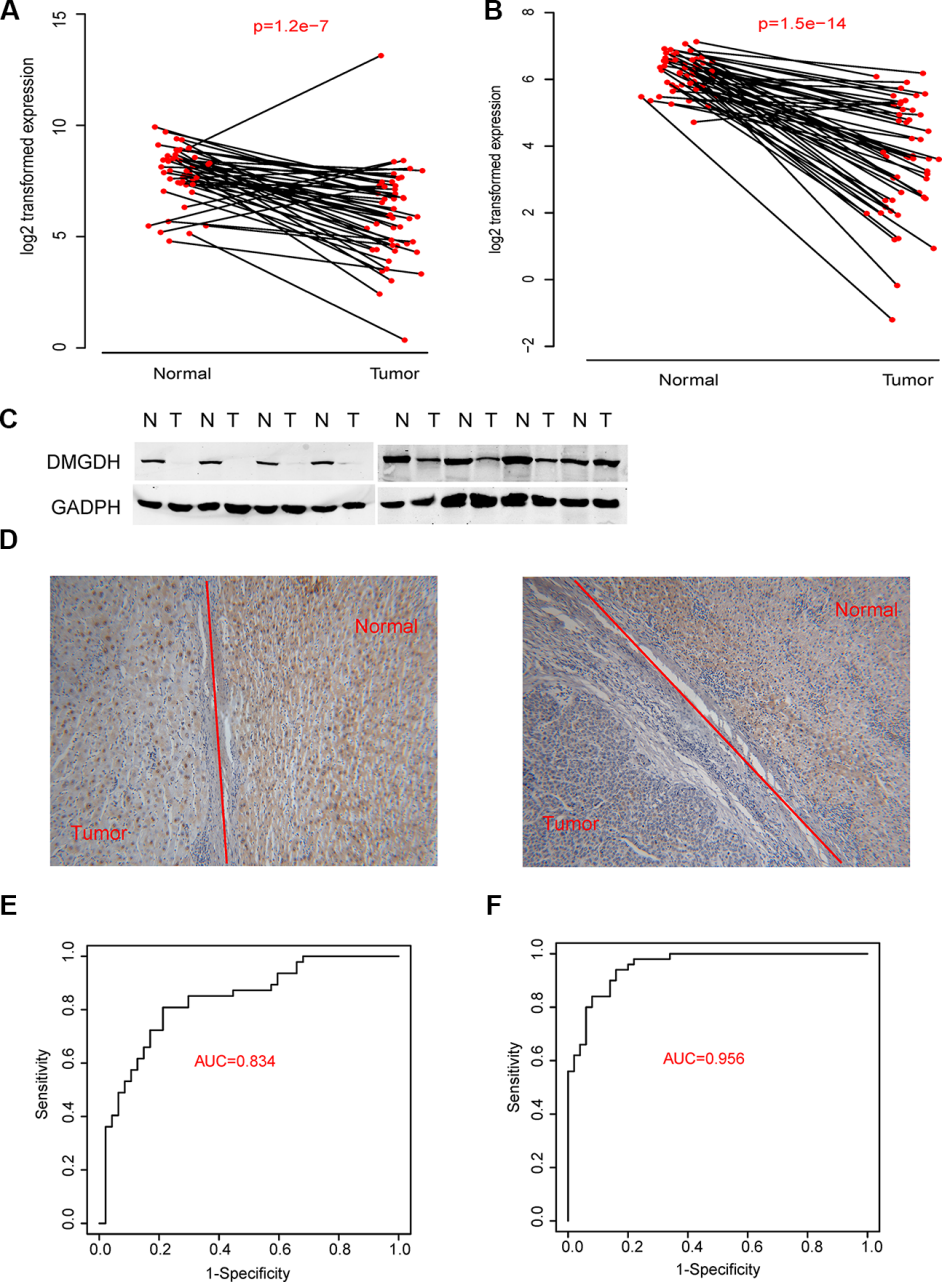
DMGDH expression level is associated with both carcinogenesis The DMGDH mRNA expression level of normal-tumor paire evaluated with (**A**) RNA-seq and (**B**) QPCR; and the DMGDH protein expression level asessed by (**C**) Western blot and (**D**) immunohistochemistry are show. The diagnostic effect of DMGDH mRNA is shownin both (**E**) QPCR and (**F**) RNA-seq dataset with receiving operating characteristic curve (ROC).

To comprehensively screen altered metabolism related genes during carcinogenesis, we performed RNA-seq on 100 samples consisting of tumor tissue and peri-tumoral tissue from 50 HCC patients. Among these patients, 13 were categorized into a high metastasis group and 14 were categorized into a low metastasis group. We evaluated the relative gene expression levels and potential somatic mutation/loss of heterogeneity of these samples. We also identified the differential gene expression between normal cells and tumor cells (approximately 8900 genes, in total), low metastasis and high metastasis tumor groups (1789 genes) (Supplementary Table S1A–S1B). In accordance with previously reported metabolic gene lists and pathways, we found that several of themwere significantly enriched [11] (Supplementary Table S1C). Considering differentially expressed genes, somatic mutations and significantly altered pathways, several genes were selected (Table 1) for further study. Of these genes, we noticed a barely reported gene, DMGDH, was down regulated in carcinogenesis (Figure 1A). The mutation and/or loss of heterogeneity rate of this gene was ~36%. We also conducted qPCR estimated on 47 samples and found that the DMGDH expression level in tumors is significantly different than normal cells (Figure 1B, detailed clinical information of the two datasets were available in Supplementary Table S1D) in 92% of samples. Western blots detecting DMGDH were also conducted on 30 tumor-normal pairs. We found that in ~80% samples, the DMGDH protein level in tumor tissue was significantly higher than that of normal tissue (Figure 1C). We also conducted immunohistochemical tests on 10 pairs of samples and found that almost all of the over expressed DMGDH is observed in these samples (Figure 1D).

**Table 1:** The significantly altered metabolic gene list

Gene	Class	Low-high metastasis fold change (log2)	Low-high metastasis FDR	Normal-Tumor fold change (log2)	Normal-Tumor FDR	Mutation/LOH rate	significant pathway
**AKR1D1**	Steroid	−1.56545	0.0006	−2.63194	0.00005	0.18	yes
**BHMT2**	Glycine	−1.56258	0.00005	−1.43584	0.00005	0.26	yes
**CPS1**	Urea	−2.56255	0.0001	−2.05089	0.00005	0.56	yes
**DMGDH**	Glycine	−1.63297	0.0002	−1.91773	0.00005	0.32	yes
**HPD**	Tyrosine	−2.84098	0.00005	−2.60153	0.00005	0.22	yes
**IDO2**	Tryptophan	−4.40572	0.00005	−4.58482	0.00005	0.08	yes

We then tested the diagnostic performance of DMGDH levels in HCC with receiving operating curve (ROC) to distinguish tumor tissue from the normal. We found that in the qPCR dataset, the area under curve (AUC) reached 0.834 (Figure 1E). The result was then validated with the RNA-seq data, and the AUC reached 0.956 (Figure 1F). These results indicate that DMGDH may be a valuable biomarker of HCC diagnosis.

### The prognostic effect of DMGDH and correlation with clinical observations

**Figure 2:**
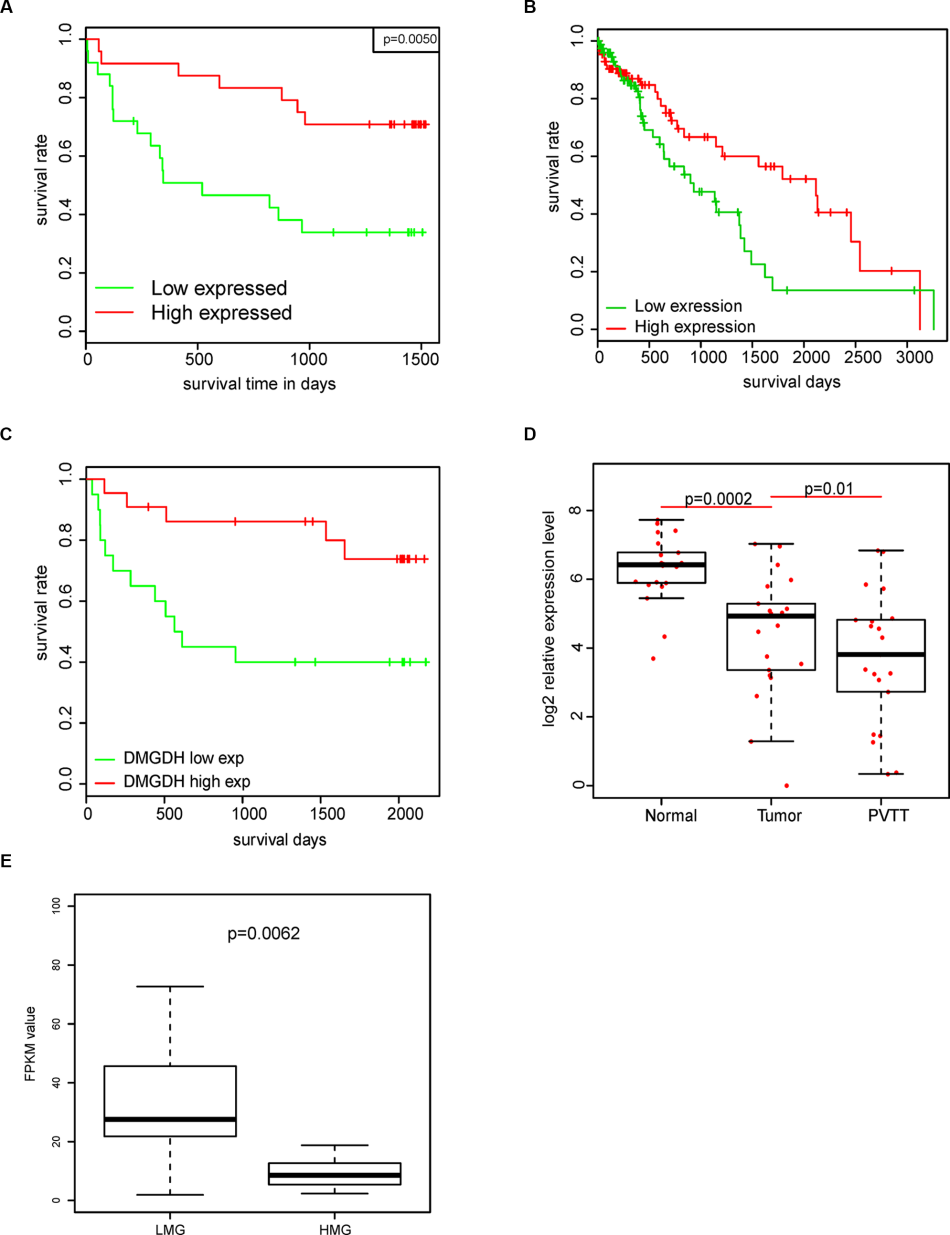
Prognostic effect of DMGDH in HCC The survival rate of DMGDH highly expressed group is significantly better than lowly expressed group in (**A**) QPCR, (**B**) RNA-seq, and (**C**) TCGA datasets. And (**D**) the mRNA expression level of PVTT is significantly lower than tumor, and tumor is lower than normal. (**E**) mRNA FPKM values (Fragments Per Kilobase of transcript per Million mapped reads) in RNA-seq data of LMG and HMG is also shown.

To further interpret the effects of DMGDH on HCC, we analyzed the survival of patients with different gene expression levels of DMGDH. The survival rate of patients with higher DMGDH levels is significantly higher than those without (Figure 2A). To avoid the over fit of our data, we also conducted survival analysis on samples from TCGA (http://cancergenome.nih.gov/) dataset, and RNA-seq dataset. The survival of patients with high DMGDH expression is also significantly better (Figure 2B–2C). Moreover, the results from 21 pairs of normal-tumor-PVTT (portal vein tumor thrombosis) showed that the mRNA and protein levels are significantly higher in PVTT cells than tumor cells, which are higher than the corresponding normal cells (Figure 2D, Supplementary Figure S1). Furthermore, in our RNA-seq data, we also found that this gene is also differentially expressed in the high metastasis group (HMG, *N* = 14) and low metastasis group (LMG, *N* = 13). The LMG group expressed DMGDH nearly 3-fold compared with the HMG group (Figure 2E).

To interpret the effect of DMGDH on prognosis and metastasis, we correlated the expression level of DMGDH in 47 samples with their clinical information. We detected that some clinical observations, including recurrence, age, differentiation level, embolus, and AFP level were significantly different between the DMGDH-high-expression (DHE) group and the low-expression (DLE) group (Table 2). The DHE group has a much better clinical observation than the LHE group.

**Table 2:** DMGDH expression associated clinical observations

variable	Low	High	*p*_value
**Age**			**0.011**
< 60	21	11	
> 60	3	12	
**Differentiation**			**0.0032**
1, 2	0	7	
3, 4	24	15	
**Stage**			0.08
1, 2	11	6	
3, 4	13	16	
**Embolus**			**0.0014**
No	7	16	
Yes	17	5	
**AFP**			**0.0012**
Low	2	13	
High	21	10	
**Daughter nodule**			0.096
No	15	20	
Yes	9	3	
**Recurrence**			**0.036**
No	5	14	
Yes	10	5	

some clinical information is missing.

All these results demonstrate that DMGDH is a metastasis suppressor gene. It correlates with survival and other important clinical observations, including embolus formation and recurrence, suggesting that DMGDH suppresses metastasis in HCC.

### The DMGDH interferes affects migration *in vitro*

**Figure 3:**
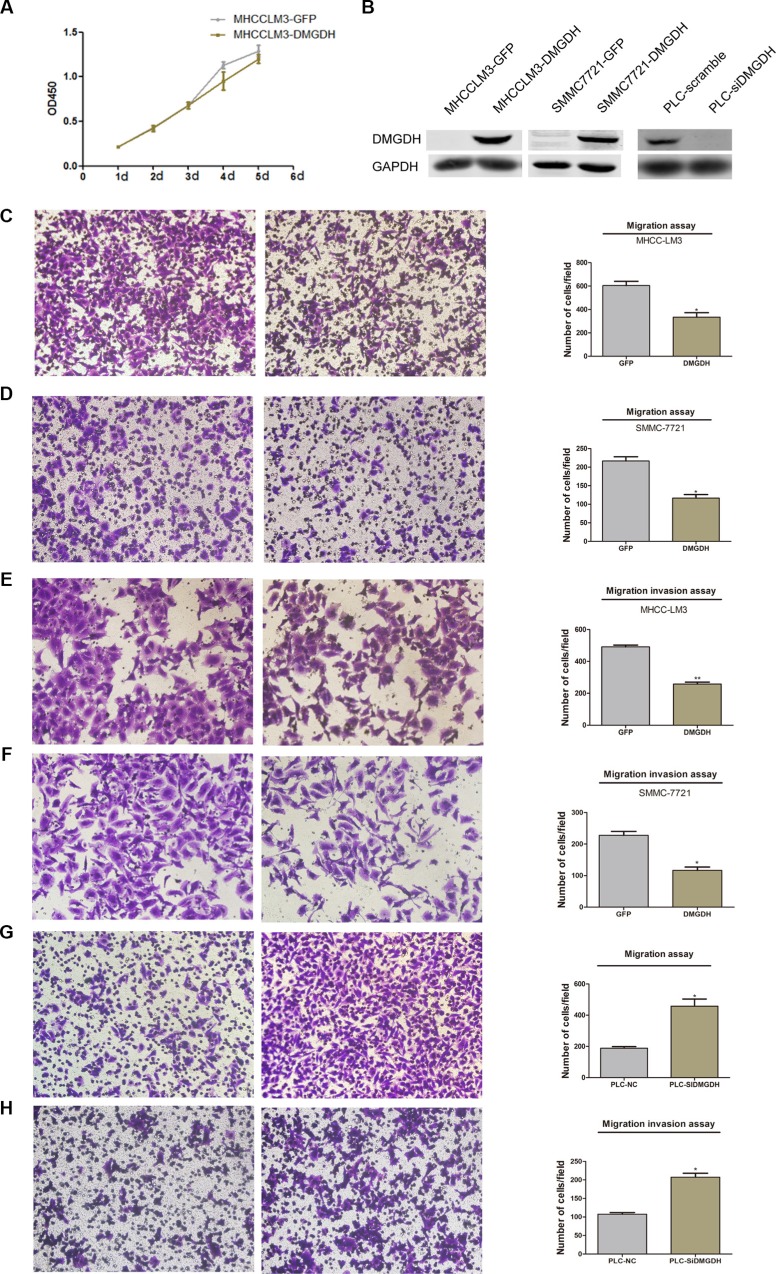
The migration suppression effect of DMGDH on HCCcell lines (**A**) The Western blot of over-expression and RNAi of cell lines. Although the proliferation is (**B**) comparable between GFP and DMGDH + GFP over expression group, after over expression of DMGDH, (**C**–**D**) the migration rate of DMGDH over expression group is significantly high than control in both MHCCLM3 and SMMC7721 cell lines. And it is also the case for invasion (**E**–**F**) assay. PLCcell line migration and invasion ability (**G**–**H**) increased rapidly after knock down of DMGDH.

To evaluate the metastasis and multiplication effects of DMGDH, we constructed cell lines, derived from SMMC7721 and MHCCLM3, which over-expressed DMGDH + GFP and GFP (named SMMC7721-DMGDH, MHCCLM3-DMGDH, SMMC7721-GFP, and MHCCLM3-GFP). We also performed RNAi on PLC cell line, and validated over-expression and RNAi by Western blotting (Figure 3A). Then, we performed proliferation assays on both cell lines with/without DMGDH over-expression/knock-down, and found that the proliferation rate of the cells highly express DMGDH was comparable with the control (Figure 3B), indicating that the reproduction rate is not affected by DMGDH.

Then, we conducted a migration and invasion assay on SMMC7721-DMGDH, SMC7721-GFP, MHCCLM3-DMGDH, and MHCCLM3-GFP cells. Significant migration and invasion differences were observed (Figure 3C–3F) between experimental and control cells. The number of cells that migrated from one side of aTrans-wellto the other side decreased by over 50% in the DMGDH over expression group (Figure 3C–3D). The invasion result also confirmed this finding (Figure 3E–3F. We performed a similar assay on DMGDH knock-down on PLC cell line, a cell line has a relative higher DMGDH expression than SMMC7721 and MHCCLM3 cell lines. We observed that the migration and invasion level of the experimental group (DMGDH knockdown) were significantly higher than those of the control group (Figure 3G–3H). We also evaluated the migration ability using a wound healing test, and the healing ability of cells with lower DMGDH expression was much better than cells with higher DMGDH expression (Supplementary Figure S1). Because the proliferation rates of the control group and DMGDH over expression group were almost identical, the migrated cell number reflects the migration ability of these cells. This indicates that DMGDH suppresses cell migration *in vitro*.

### Over expression of DMGDH suppresses metastasis *in vivo*

**Figure 4:**
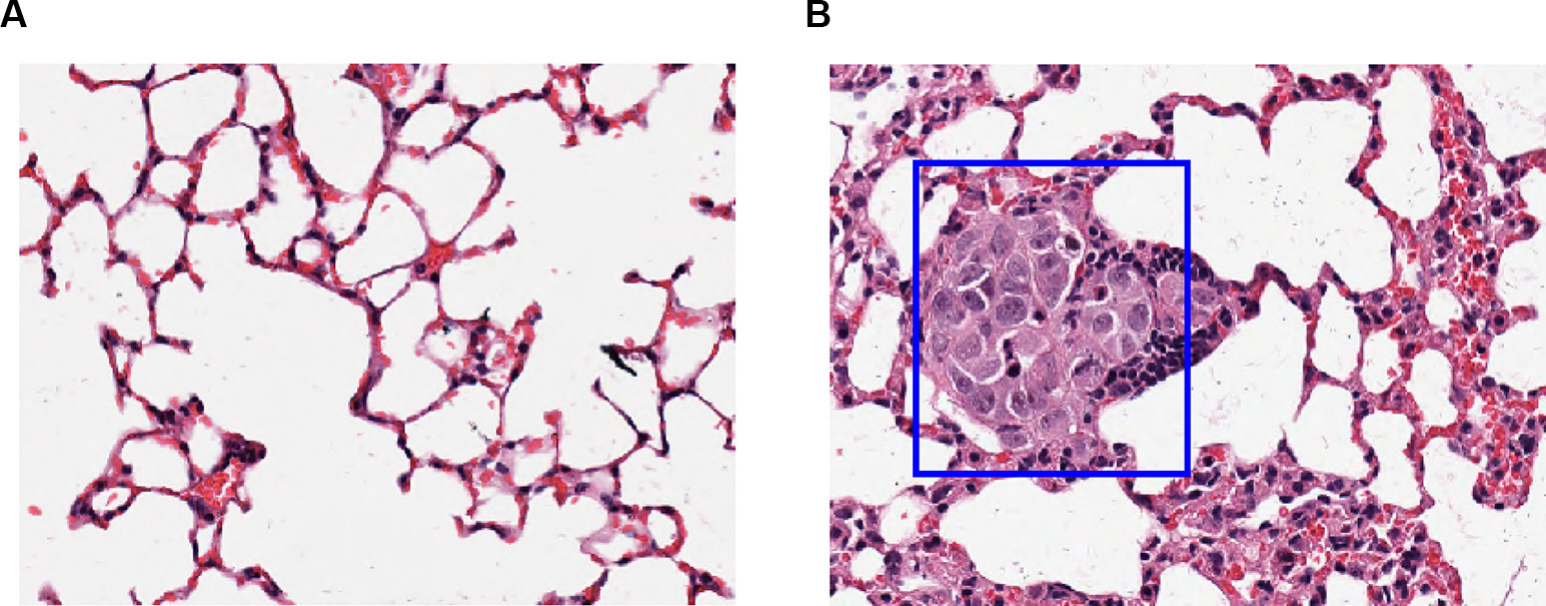
The lung histological section of mice injected with GFP/DMGDH+GFP over expression cell line (**A**) Seven out of 8 mice with DMGDH overexpressed cell line injected did not detected lung metastasis, (**B**) while 6 out of 8 mice in the control group detected lung metastasis.

To investigate whether the suppression of migration of DMGDH is also maintained *in vivo*, we constructed a mouse lung metastasis model. We injected equal numbers of MHCCLM3-GFP and MHCCLM3-DMGDH cells in naked mouse tail vein. After 4 months, the lungs were dissected and stained with hematoxylin & eosin (H&E). Injected cancer cells were detected in the lungs of 6 out of 8 mice in the MHCCLM3-GFP group, while there is only one mouse occurred cells in MHCCLM3-DMGDH group (*p* = 0.041, Figure 4). These results indicate that DMGDH also significantly suppresses migration of hepatocellular carcinoma cells *in vivo*.

### DMGDH suppresses PI3K/Akt pathway

**Figure 5:**
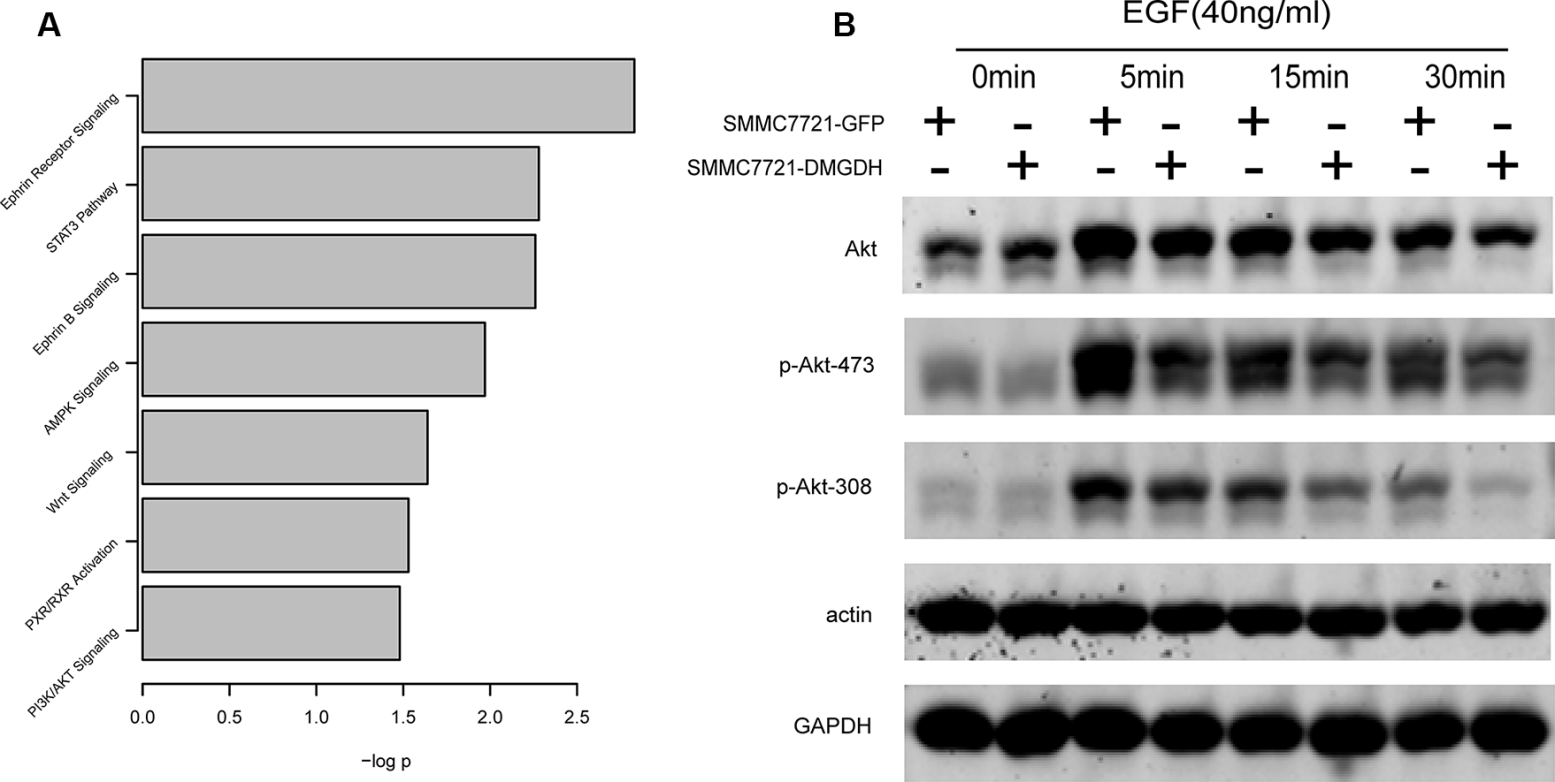
Altered pathway identification With gene expression evaluation with microarray, differentially expressed genes were detected, and (**A**) pathway analysis of these genes was performed with IPA. Among these pathways, we found that phosphorylation level of (**B**) Akt 308T and 473S is less in DMGDH over expression cell line.

Although the role of DMGDH suppression of tumor motility both *in vivo* and *in vitro* was validated by our previous experiments, the mechanisms underlying the suppression were still unclear. To more comprehensively evaluate the effect of DMGDH and the potential target of DMGDH, we analyzed the gene expression levelsinSMMC7721-DMGDH and SMMC7721-GFP cells by microarray with 3 replicates each. In total, 269 differentially expressed genes were detected (Supplementary Table S3). We analyzed these genes with IPA, and found several pathways were significantly enriched. Among these pathways, the STAT3, AMPK, WNT, and PI3K/Akt (Figure 5A) pathwaysare frequently reported in carcinogenesis and metastasis, including HCC [12–16].

We performed western blots to detect the phosphorylation of key proteins in these pathways. We found that the phosphorylation of Akt on residues 308 and 473 was significantly lower in the DMGDH over-expression group after stimulation with epithelial growth factor (EGF) (Figure 5B). Akt-308/473 phosphorylation is involved in well-known and canonical cancer invasion pathways. In summary, our results indicate that DMGDH suppresses metastasis through inhibiting the Akt signaling pathway.

## DISCUSSION

Metabolism disorders have been reported in many cancer types, including HCC [17, 18]. We integrate the gene expression, mutation/loss of heterogeneity and metabolic pathways, and identified DMGDH, a rarely reported gene, was altered in all these biological levels. We then found that the AUC reached 0.834 and 0.954 in the qPCR and RNA-seq datasets, indicating that DMGDH is a potential valuable biomarker for diagnosis; the higher DMGDH expression level correlated with better clinical observation. These results indicate that it is also a good prognostic marker for HCC.

Although the mechanism DMGDH suppresses migration keeps unknown, we detected that WNT, STAT3, and PI3K/AKT pathways alterd in DMGDH over expressed cells. And the phosphorylation ofp-308T-Akt and p-473S-Akt was inhibited in presence of DMGDH. Phosphorylation of these sites is well-known to induce Akt activation [19]. Anactivated Akt pathway is a canonical metastasis marker in many cancers [20], and inducesepithelial-mesenchymal transition (EMT) by inhibiting GSK-3β, leading to the stabilization and nuclear localization of Snail, thereby triggering cell migration and EMT [21].

In summary, we detected a novel tumor suppressor gene, DMGDH, as a biomarker that is capable of distinguishing between normal and tumor tissue, and this gene also suppresses metastasis *in vitro*, *in vivo*, and in clinical observations. Its inhibition effect is at least partly due to its repression of Akt activation.

## MATERIALS AND METHODS

### RNA-seq data analysis

The RNA extraction was performed with TRIzol reagent (Invitrogen, CA) following the manufacture provided protocol. Written informed consents were obtained from patients and the study was approved by ethnic committee Eastern Hepatobiliary Surgery Institute. RNA-seq procedure was conducted under the instruction of standard Illumina sequencing. The RNA-seq raw data were analyzed for quality control with the fastx-toolkit (http://hannonlab.cshl.edu/fastx_toolkit) and the fast-QC (http://www.bioinformatics.babraham.ac.uk/projects/fastqc/) programs. The trimmed quality threshold was set to 20, and the minimum length was designated as 10. The trimmed reads were subsequently re-matched. Then, the reads were mapped to the human genome, Hg19, and the transcriptome from UCSC [22] by TopHat software [23] using the default parameters. The relative gene expression levels (FPKM, fragments per kilobase per million mapped fragments) were evaluated by the Cufflinks program [24] using the default parameters, and “cuffnorm” was used for normalization of the gene expression level of these samples. The differentially expressed genes were determined by the command line “cuffdiff” in the cufflinks program. The somatic mutation analysis was conducted by samtools [25] and Varscan [26] with the aligned reads. The mutated/LOH sites were annotated with annovar software [27]. The raw and processed data of RNA-seq is now available on GEO (GSE77314).

### Extraction of RNA, preparation of cDNA, and quantitative real-time PCR (qRT-PCR)

Total RNA from different cell lines and HCC samples was isolated using TRIzol reagent (Invitrogen, CA) following the manufacturer's instructions. The quality of the total RNA was assessed by a Nanodrop 2000 and agarose gel electrophoresis. First-strand cDNA was synthesized from 1–2 μg of total RNA using random primers and M-MLV Reverse Transcriptase (Invitrogen, CA). Real-time PCR was performed according to the SYBR Green kit (Applied TaKaRa, Japan) in an ABI PRISM 7900 sequence detector (Applied Biosystems, Carlsbad, CA) with 18s as the endogenous control. The relative mRNA levels were calculated based on the Ct values and normalized according to the 18s expression.

### Western blot analysis

Total protein was extracted from cell or HCC tumor specimens with RIPA Lysis Buffer and PMSF (Beyotime Co., China) according to the manufacturer's instructions, and centrifuged at 12,000 g for 15 minutes. Protein concentrations were measured using the bicinchoninic acid assay. Antibody dilutions were 1:500 for the DMGDH polyclonal antibody (Proteintech Group, Inc., Chicago, USA) and 1:10000 for GAPDH (Santa Cruz Biotechnology). Antibody binding was detected with an Odyssey infrared scanner (Li-Cor Biosciences, Inc.)

### Immunohistochemistry

Immunohistochemistry was performed on the HCC samples with a two-step immunoperoxidase technique. The DMGDH polyclonal antibody (Novus, USA) diluted 1:50 was used as the primary antibody. Briefly, after heating the sections in 10 mmol/L citrate buffer for antigen retrieval, the sections were incubated first with the primary antibody, and then with the secondary antibody for an hour at room temperature. Finally, the sections were developed in diaminobenzidine solution under a microscope, and counter-stained with hematoxylin.

### *In vitro* cell-behavior assays

For the wound-healing assays, monolayers of cells plated in 12-well plates were wounded by scraping with a 200 μL plastic pipette tip and then rinsed several times with medium to remove anyfloating cells. The wound-healing process was monitored with an inverted light microscope (Olympus, Tokyo, Japan). For migration and invasion assays, Transwell filter champers (Costar, Corning, NY) and BioCoat Matrigel invasion chambers (BD Biosciences) were used according to the manufacturers’ instructions. Six random microscopic fields were counted per field for each group, and these experiments were repeated at least 3 independent times. For the cell-proliferation assays, MHCC-LM3-DMGDH and control cells (3 × 10^3^ cells/well) were seeded in 100 μL of growth medium in 96-well plates for various time periods. Cell proliferation was evaluated by measuring cell viability with the Cell Counting Kit 8 assay (Dojindo Laboratories, Kumamoto, Japan) according to manufacturer's instructions.

### *In vivo* metastasis

Sixteen 6-week-old male nude mice were randomized into 2 groups (*N* = 8, for each group) and either MHCC-LM3-DMGDH or MHCC-LM3-GFP cells (4 × 10^5)^ were injected into the tail vein for the pulmonary metastatic model. Mice were sacrificed at 16 weeks post injection and examined microscopically by H & E staining for the development of lung metastatic foci. Animals were housed in cages under standard conditions, following the requirements of the Second Military Medical University Animal Care Facility and the National Institutes of Health guidelines.

### Microarray data analysis

RNA was extracted from both MHCCLM3-DMGDH and MHCCLM3-GFP cell lines with 3 replicates each. Evaluation of gene expression levels was performed with Agilent Whole Human Genome Oligo Microarray (4 × 44 K). The operation was conducted by Shanghai Biotechnology Corporation. After quality control, background corrections and normalizations were performed with Feature Extraction software 10.7 (Agilent technologies, Santa Clara, CA, US) and the Quantile algorithm, GeneSpring Software 12.6.1 (Agilent technologies, Santa Clara, CA, US). Genes differentially expressed between MHCCLM3-DMGDH and MHCCLM3-GFP were identified according to *p* values (*p* < 0.001) and log transformed fold changes (< −0.2 or > 0.2). The raw and processed data is available on GEO (GSE77329).

## SUPPLEMENTARY MATERIAL FIGURES AND TABLES




