# Supplementary material for: Potential diagnostic and prognostic marker dimethylglycine dehydrogenase (DMGDH) suppresses hepatocellular carcinoma metastasis in vitro and in vivo
Source: Oncotarget. 2016 Apr 22;7(22):32607–16. doi: 10.18632/oncotarget.8927 (PMC5078037; doi:10.18632/oncotarget.8927)
Supplement: Supplementary file 1 [file oncotarget-07-32607-s001.pdf]

Potential diagnostic and prognostic marker dimethylglycine dehydrogenase (DMGDH) suppresses hepatocellular carcinoma metastasis *in vitro* and *in vivo*

Supplementary Materials

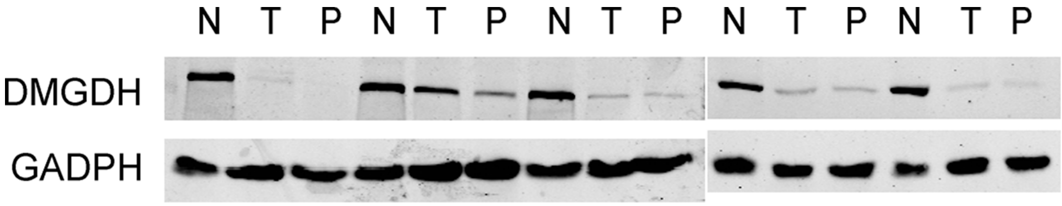

Supplementary Figure S1: Protein level of normal-tumor-PVTT pair evaluated by Western blot.

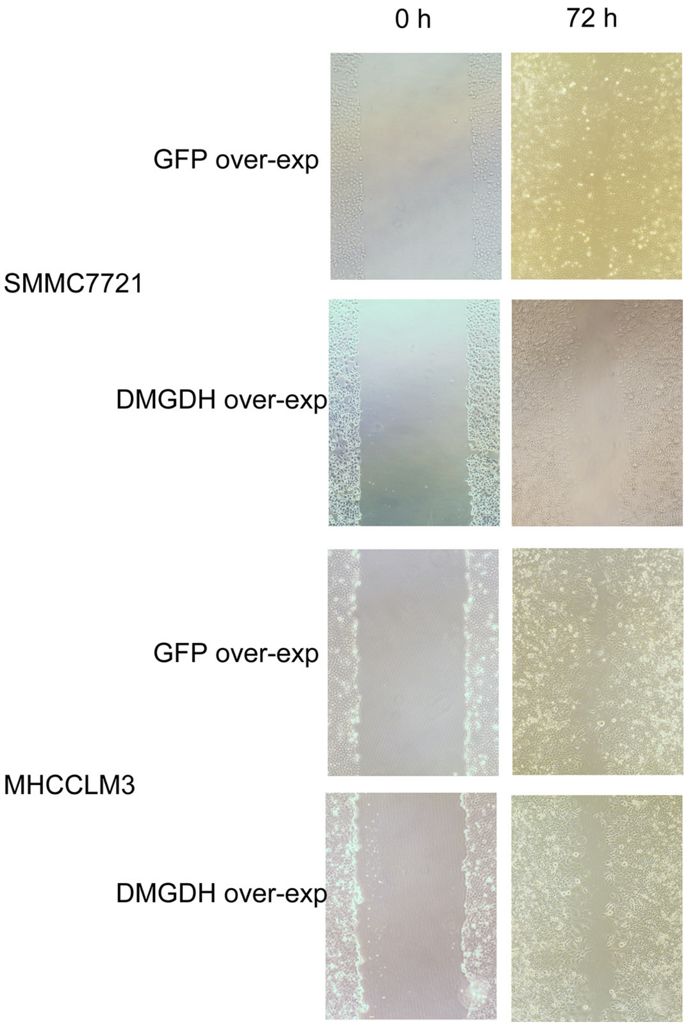

Supplementary Figure S2: Would healing test of DMGDH+GFP and GFP over expressed cells in MHCCLM3 and SMMC7721 cell line.

Supplementary Table S1A: Differentiall expressed genes (DEG) between normal and tumor identified by RNA-seq

Supplementary Table S1B: DEG between highly metastasis group and lowly metastasis group

Supplementary Table S1C: Significantly altered metabolic pathways according to DEG

Supplementary Table S1D: Clinical information of RNA-seq and QPCR datasets

Supplementary Table S3: DEG between DMGDH+GFP- and GFP- over expression cells evaluated by microarray
